# Knowledge about Iodine in Pregnant and Lactating Women in the Oslo Area, Norway

**DOI:** 10.3390/nu9050493

**Published:** 2017-05-13

**Authors:** Lisa Garnweidner-Holme, Inger Aakre, Anne Marie Lilleengen, Anne Lise Brantsæter, Sigrun Henjum

**Affiliations:** 1 Department of Nursing and Health Promotion, Faculty of Health Sciences, Oslo and Akershus University College of Applied Sciences, 0130 Oslo, Norway; inger.aakre@hioa.no (I.A.); anne.marie.lilleengen@hioa.no (A.M.L.); sigrun.henjum@hioa.no (S.H.); 2 Department of Environmental Exposure and Epidemiology, Norwegian Institute of Public Health, 0403 Oslo, Norway; annelise.brantsaeter@fhi.no

**Keywords:** iodine, knowledge, iodine status, pregnancy, lactation

## Abstract

Background: Lack of knowledge about iodine may be a risk factor for iodine deficiency in pregnant and lactating women. The aim of this study was to assess knowledge about iodine and predictors of iodine knowledge scores among pregnant and lactating women. The study also examined whether iodine knowledge scores were associated with iodine status. Methods: A cross-sectional study was performed on 804 pregnant women and 175 lactating women from 18 to 44 years of age in 2016 in the Oslo area, Norway. Knowledge about iodine was collected through a self-administered, paper-based questionnaire. Iodine concentrations in urine and breast milk were measured using an inductively coupled plasma mass spectrometer (ICPMS). Results: 74% of the pregnant women and 55% of the lactating women achieved none to low iodine knowledge scores. Higher educated pregnant women and those who had received information about iodine had significantly higher knowledge scores. In lactating women, increased age was associated with higher knowledge scores. Knowledge scores were not associated with participants’ iodine status. Conclusion: This study revealed a lack of knowledge about the importance of iodine in pregnant and lactating women, as well as about the most important dietary sources. Public education initiatives are required to increase the awareness about iodine in these population groups.

## 1. Introduction

Dietary iodine is essential for the production of thyroid hormones, which are crucial for normal growth and neurodevelopement in utero [1,2,3,4]. Due to increased thyroid hormone production, increased renal iodine losses, and fetal iodine requirements, pregnant and lactating women’s iodine requirements are increased by ≥50% [5,6].

Health authorities in Nordic countries recommend 175 µg/day for pregnant and 200 µg/day for lactating women [7]. According to the World Health Organization (WHO), the median urinary iodine concentration (UIC) is the best marker for assessing iodine status in pregnant women, since >90% of the dietary iodine eventually appears in the urine [8,9]. Iodine deficiency is a major public health concern and has been described as the single greatest cause of preventable mental impairment [10]. Severe deficiency during pregnancy has the most serious effect and can result in cretinism. Mild-to-moderate iodine deficiency may cause maternal and fetal hypothyroidism and impair neurological development of the fetus [11,12,13]. Europe is the WHO region with the highest percentage of pregnant women with iodine deficiency. Two-thirds of the European countries that have assessed iodine nutrition during pregnancy reported inadequate iodine intakes [14]. There are indications that pregnant and lactating women are mildly iodine-deficient in several Nordic countries [15,16]. Norway has been classified as mildly iodine-deficient [12]. Currently, the largest study of iodine intake in the world was the Norwegian Mother and Child Cohort Study (MoBa). Of 61,904 women, 16% had an iodine intake of <100 µg/day, 54% had iodine intake below the Nordic recommendation of 175 µg/day, and only 22% reached the recommendation of 250 µg/day by the WHO, the United Nations Children’s Fund (UNICEF) and the International Council for Control of Iodine Deficiency Disorders (ICCIDD) [17]. The use of supplements containing iodine was reported by 31.6% of mothers-to-be. The primary source of iodine from food was dairy products, contributing 43% of daily iodine intake in iodine-supplement users and 67% of daily iodine intake in those who did not use supplements. The median intake of iodine from food was 141 µg/day, and the additional contribution from supplements in iodine supplement users was 107 µg/day. The insufficient iodine intake levels shown in pregnant women have set alarm bells ringing at the Norwegian Directorate of Health, and an Iodine Committee is now trying to assess the situation and has been commissioned to present proposals for the alleviation of iodine deficiency in vulnerable groups [15]. There is a large knowledge gap regarding the iodine status during lactation in most Nordic countries, and the only country with available data is Denmark [15].

Information about iodine during pregnancy and lactation may be important to increase women’s knowledge and to prevent suboptimal iodine intake. In Norway, health professionals at Mother and Child Health Centers (MCHCs) play a unique role in the provision of dietary information. They increasingly provide antenatal care as well as health services for practically all lactating women [18]. In the Norwegian guidelines for antenatal care, health professionals are encouraged to provide women with basic information about a healthy diet, without specific focus on iodine [19]. Previous studies suggest that a lack of information and knowledge about iodine may be a major risk factor for iodine deficiency in pregnant and lactating women [20,21,22,23,24]. In these studies, women had difficulties identifying important dietary sources of iodine as well as adverse health outcomes related to iodine deficiency. For instance, only half of the participants in cross-sectional studies among pregnant women in Australia correctly identified seafood as a good source of iodine and more than half of the participants could not identify any adverse health problem related to iodine deficiency [20,25]. Women’s age and educational level may influence their knowledge about iodine [23]. However, studies about associations between pregnant and lactating women’s knowledge and iodine status are sparse. Thus, the aim of the present study was to describe pregnant and lactating women’s knowledge about iodine and to explore possible predictors of their iodine knowledge scores. The study also examined whether iodine knowledge scores were associated with women’s iodine status. To our knowledge, this is the first study to assess the knowledge about iodine in pregnant and lactating women in Norway.

## 2. Materials and Methods

This cross-sectional study included 804 pregnant and 175 lactating women recruited from February to December 2016 at 12 MCHCs Oslo area, Norway. To be eligible for the study, participants had to be able to read and write in Norwegian. Women were recruited by midwives and public health nurses at the MCHCs. Recruiters were asked to provide participants with routine care, without specific focus on iodine. In total, 254 lactating women were invited to participate, 193 accepted, and 175 (91%) completed the questionnaire. Concerning pregnant women, 812 chose to participate, and 804 (99%) fulfilled the questionnaire.

Data were collected anonymously through a paper-based, self-reported questionnaire. Seven questions from the questionnaire were used for this study. Questions to determine participants’ knowledge scores were as follows: (1) Do you know what iodine is? (2) What are the most important dietary sources of iodine? (3) Why is iodine important? (4) What do you know about the current iodine status among pregnant women in Norway. In addition, participants were asked: (5) Do you feel confident that you achieve your daily requirements for iodine? and (6) Did you receive information about iodine from health professionals during pregnancy.

These questions were chosen from previous studies about iodine knowledge [20,22,26] and adopted after a content validation by three subject experts and a pilot test among four pregnant women. Background information included women’s age, week of pregnancy, previous pregnancies, pre-pregnancy height and weight, educational level, and smoking habits. Participants were also asked about their country of birth, how long they have lived in Norway, and what language they speak at home.

Spot urine samples were collected to determine UIC among pregnant and lactating women, and breastmilk samples to determine breast milk iodine concentration (BMIC). For more details, see [27] (manuscript in preparation). Urine and breast milk samples were analyzed for iodine coupled plasma-mass spectrometry method (ICP-MS) at the Norwegian University of Life Sciences. UIC and BMIC were collected at the same time the participants answered the questionnaire. Information about gestational week was self-reported.

Data were analyzed using IBM SPSS version 24 (IBM Corp., Armonk, NY, USA). Iodine knowledge variables were used to calculate a knowledge score used for descriptive purposes. Correct answers generated 2 points, correctly identified false answers generated 1 point, and incorrect answers gave 0 points. Based on the knowledge scores from each of the knowledge questions, a total knowledge score was assessed ranging from 0 to 24, and categorized as follows: no knowledge (0 point), poor knowledge (1–6 points), low knowledge (7–12 points), medium knowledge (13–18 points), and high knowledge (19–24 points). The total iodine score was tested for differences between pregnant and lactating women with a Mann-Whitney U test.

Due to a lack of validated cut-off regarding knowledge scores, the total scores were categorized at the 66th percentile for the statistical analyses, which was decided prior to the analyses. Scores below the 66th percentile were categorized as medium to low knowledge, while scores above the 66th percentile were categorized as high knowledge. Logistic regression analyses were used to assess the associations between knowledge scores and possible determinants: age, education (0 = completed high school or lower education, 1 = higher education), number of children, Human Development Index (HDI) of mother country (0 = low/medium/high HDI, 1 = very high HDI), information about iodine during pregnancy (0 = none/don’t remember, 1 = yes), and tobacco habits (0 = use tobacco/did use before pregnancy tobacco, 1 = don’t use tobacco) among pregnant and lactating women. Differences in iodine status, indicated by UIC and BMIC between the highest tertile of the knowledge score and the lowest two tertiles were tested with a Mann–Whitney U test. Associations between UIC, BMIC, and knowledge scores were also tested with multiple linear regression models. UIC and BMIC were log (2) transformed due to skewed distribution. The models were adjusted for age and body mass index (BMI). Residuals that were ± 3 were removed from the models. 

The study was conducted according to the guidelines laid down in the Declaration of Helsinki [28] and was approved by the Regional Committee for Medical and Health Research Ethics Norway (Nr.2015/1845). Participants gave their written consent and received written information about the study.

## 3. Results

Sample characteristics are shown in Table 1. Approximately 16.6% of the pregnant women and 7.4% of the lactating women had received information about iodine from their health professionals, whereas 17.6% of the pregnant women and 12.6% of the lactating women could not remember whether or not they had received information.

### 3.1. Knowledge and Confidence in Terms of Achieving Daily Recommendations for Iodine

Table 2 shows that more than half of the pregnant women (51.5%) and lactating women (58.9%) reported knowing what iodine is; while 23.4% of the pregnant women and 34.3% of the lactating women could not remember. About 60% of the pregnant women (60.9%) and lactating women (58.3%) did not know whether or not they received enough iodine through their diet. Only 5.0% of the pregnant women and 37.7% of the lactating women felt confident that they had achieved the daily recommendations for iodine through their diet.

Data regarding iodine knowledge applied to determine knowledge scores are presented in Table 3. Around half of the lactating women correctly identified milk (54.3%) and fish and seafood (57.7%) as the most important dietary iodine sources. Dietary supplements were only chosen by 12.6% of the lactating women. Pregnant women’s knowledge about the most important dietary iodine sources was lower with milk (28.9%), fish and seafood (37.1%) and dietary supplements (7.6%). Some foods were incorrectly identified as the most important sources of iodine, including meat (15.7% in pregnant women; 13.7% in lactating women), fruit (10.6% in pregnant women; 16.0% in lactating women), and bread (12.7% in pregnant women; 16.6% in lactating women). More than one-third of the pregnant women (37.4%) and 17.7% in the lactating women did not know what foods (from the list) were the most important sources of iodine. Lactating women had more knowledge about the importance of iodine than pregnant women; 45.7% correctly identified iodine intake as important for normal child growth and development, 17.1% for normal fetal development, and 48.0% for maintaining normal metabolism. Among pregnant women, these figures were 24.3%, 16.3%, and 26.6%, respectively. Only about 20% of pregnant and lactating women thought that iodine intake that is too low is a current public health problem in Norway, whereas around 70% were unsure.

The total iodine knowledge scores are presented in Table 4. The median (p25–p75) score was 7 (0–13) and 12 (6–15) for pregnant and lactating women, respectively, on a scale from 0 to 24. Pregnant women had a significantly lower score than did lactating women (*p* < 0.001); 74.5% of the pregnant women and 54.9% of the lactating women had an iodine knowledge score that was nil or low.

Associations for being in the highest tertile of the knowledge scores among pregnant and lactating women are found in Table 5. For pregnant women, having a higher education yielded a significantly and substantially increased probability of being in the upper tertile of the knowledge score, showing an adjusted OR (95% CI) of 1.54 (1.12, 2.15). Whether the women had received information regarding iodine during pregnancy also significantly increased the probability of being in the upper tertile of the knowledge score with an adjusted OR (95% CI) of 2.87 (1.95, 4.23). Among lactating women, age had significant associations with knowledge score, whereas increased age yielded higher probability of being in the highest tertile of the knowledge score with an adjusted OR (95% CI) of 1.14 (1.04, 1.25). Information about iodine yielded a substantially increased probability of a higher iodine knowledge score among lactating women; however, this effect was not significant.

### 3.2. Knowledge Scores and Iodine Status

There were no significant differences in UIC or BMIC between pregnant and lactating women with knowledge scores above the 66th percentile and the women with knowledge scores below the 66th percentile (Table 6). For lactating women, the mean BMIC and its variation was higher among women with the highest knowledge score, but not significantly. UIC was quite evenly distributed in the two groups both for pregnant and for lactating women. Associations between participants’ knowledge score and iodine status were also tested with multiple regression models, adjusted for age and BMI (data not shown). The knowledge score was not significantly associated with a UIC for either pregnant or lactating women, showing adjusted coefficients (95% CI) of 0.002 (−0.14, 0.14), 0.015 (−0.35, 0.38), respectively, nor was there an association with BMIC for lactating women, showing an adjusted coefficient (95% CI) of 0.25 (−0.04, 0.54). However, for lactating women, there was a trend indicating that women in the highest tertile of the iodine knowledge score had a higher BMIC (*p* = 0.093).

## 4. Discussion

This study found a lack of knowledge about iodine in pregnant and lactating women. Previous studies in New Zealand, the United Kingdom, and Australia have also found that pregnant and lactating women have little knowledge about iodine [20,23,25]. In line with these studies, participants had difficulties identifying the most important dietary iodine sources. The results for the correct dietary sources of iodine in the present study, such as milk and fish and seafood are similar to other studies [20,23]. However, 41% of the pregnant women and 46% of the lactating women chose iodized salt as one of the most important dietary sources of iodine. It should be noted that iodized salt is a negligible source in Norway. The fortification of salt with iodine is voluntary, and very few brands contain iodine. The permitted level is 5 µg iodine/g salt, which is too low to impact the iodine intake of those who use this salt. The food industry is not allowed to use iodine-fortified salt in Norway [15]. More than half of the pregnant women (53%) and about one-third of the lactating women (31%) in our study did not know what iodine is important for. These numbers are also comparable with a study among 520 females of childbearing age in the United Kingdom and Ireland, where almost half (41.0%) of the participants did not know or could not correctly identify any health problem associated with iodine deficiency. Studies among women of childbearing age in New Zealand, the United Kingdom, and Australia found little awareness of the fact that iodine deficiency is a current public health concern [20,23,26], which was also found in our study. In comparing the results from these studies, it is important to keep in mind that there has been little focus on iodine in the last several years in Norway. Compared to Australia and New Zealand, there have not yet been initiatives from the government to improve iodine status.

The present study found significantly lower knowledge scores among pregnant women than in lactating women. Few other studies included both pregnant and lactating women. Charlton et al. did not find differences between pregnant and lactating women’s knowledge about iodine [29]. A previous qualitative study found that pregnant women received little nutrition-related information in antenatal care in Norway. Basic information about a healthy diet was often first provided towards the end of pregnancy [30]. This might explain the lower knowledge about iodine in pregnant women compared to lactating women, as almost half of the pregnant women (47%) answered the questionnaire before the last trimester and might not yet have received information about iodine. We found that pregnant women who received information about iodine had higher knowledge scores. However, the majority of pregnant women did not receive information about iodine. A lack of information about iodine during pregnancy has also been reported in studies from Australia, New Zealand, and the UK [23,24,26]. Pregnant women are considered to be more receptive to nutrition-related information than are non-pregnant women [31,32]. As previously mentioned, health professionals providing antenatal care in Norway are encouraged to provide women with general information about a healthy diet. Even though pregnant and lactating women may be at risk for suboptimal iodine intake, there are currently no public education initiatives to improve the situation. Given the importance of an optimal iodine status during pregnancy and our finding that information was associated with higher knowledge scores, pregnant women should be provided with specific information about iodine at the beginning of the pregnancy.

Even though information is crucial to improve pregnant and lactating women’s knowledge about iodine, it is important to consider other factors that predict women’s knowledge about iodine. In our study, being highly educated was a predictor for higher knowledge scores in pregnant women, whereas older lactating women had higher knowledge scores. Other studies on possible predictors of women’s knowledge about iodine show divergent results [23,26]. Lucas et al. found poor knowledge about iodine in 142 Australian pregnant women independently of age, education, and parity, whereas highly educated women in the United Kingdom and Ireland had greater awareness of iodine. Contrarily to our study, younger women (18–25 years) had higher knowledge about iodine [23]. According to a national survey about breastfeeding rates from the Norwegian Directorate of Health, the amount of breastfeeding women in Norway (either exclusively or partially) ranges from 94–81% from 1 to 4 months of the child’s age. Lactating women were more highly educated compared to the female population at large [33], which might explain why education was not a predictor of knowledge in our study. Given these divergent results, more studies about the predictors of women’s knowledge about iodine are necessary in order to target education initiatives to increase pregnant and lactating women’s awareness of iodine.

A lack of nutritional knowledge is often associated with less healthy dietary habits [34]. However, participants’ knowledge scores were not associated with women’s iodine status. This finding is in accordance with a cross-sectional study among women in Tehran, where suboptimal iodine intake was attributed to inappropriate practices, but not to knowledge [35]. Contrarily, other studies found that a poor knowledge about iodine may be a major risk factor for iodine deficiency in pregnant and lactating women [20,21,22,23,24,36]. O’Kane et al. found that knowledge about iodine was positively associated with dietary iodine intake among women of childbearing age in the UK and Ireland. The author concluded that poor knowledge of dietary iodine sources helps explain why almost half of the participants had a daily iodine intake below the recommended nutrient intake. Zarghani et al. evaluated whether an educational intervention can improve iodine status in pregnant women in Iran [37]. They conducted a randomized controlled trial where the intervention group received a four-month educational program including face-to-face sessions and educational pamphlets in the second and third trimester of the pregnancy. Even though participants’ iodine status did not improve during the study, women gained knowledge and adopted practices to achieve their daily recommendations. It has long been recognized that failure to inform the public on iodine-deficiency disorders is one of the reasons why many intervention programs have been unsuccessful [38,39]. Our study revealed major knowledge gaps about the importance of and dietary sources of iodine that need to be addressed to prevent suboptimal intake in these population groups in the current absence of public interventions to increase iodine intake.

There are a number of considerations to be taken into account when interpreting the present study’s results. The study group was highly educated compared to the average among women in Norway and might not be representative of the entire pregnant and lactating population in Norway. In the study sample, 80% of the pregnant women and 82% of the lactation women had higher education, whereas the average in the Norwegian female population is 36% [40]. With no formally validated cut-offs for the iodine knowledge score, we used the score categorized on the 66th percentile. Although our data may be used to examine predictors of iodine knowledge and associations between iodine knowledge and iodine status, further validation of the questionnaire is needed in order to determine the specific scores of iodine knowledge. UIC was measured in spot urine samples, which is known to have large intra-individual variability [41]. It is also important to acknowledge that BMIC is a more accurate biomarker of iodine status than UIC in lactating women [42].

## 5. Conclusions

Despite growing concern regarding iodine status in pregnant and lactating women in Norway, the present study revealed a lack of knowledge about the importance of iodine in pregnant and lactating women. In addition, we found a lack of knowledge about the most important dietary sources with which daily requirements of iodine can be achieved. Even though knowledge scores were not associated with participants’ iodine statuses, it is likely that public education initiatives to improve the knowledge about iodine in this population groups will increase their awareness of iodine. The different predictors of iodine knowledge found in this study may be used to target public education initiatives.

## Figures and Tables

**Table 1:** Sample characteristics and received information about iodine ^a^.

Characteristics	Pregnant Women (*n* = 804)	Lactating Women (*n* = 175)
Age, years	31.1 ± 4.4	31.8 ± 4.2
Height, cm	167.1 ± 6.3	166.8 ± 7.1
Weight, kg	75.6 ± 13.0	68.4 ± 13.3
BMI, kg/m^2 b^	26.3 (23.9–29.3)	23.5 (21.4–26.4)
<18.5	0	3 (1.7)
18.5–24.9	287 (36.6)	105 (60.0)
≥25–29.9	335 (42.7)	47 (26.9)
≥30	162 (20.7)	20 (11.4)
Number of previous children	0.6 ± 0.7	0.6 ± 0.9
Gestational week ^c^	29.9 (24.0–36.0)	-
First trimester	28 (3.5)	-
Second trimester	344 (43.1)	-
Third trimester	426 (53.4)	-
Age of child, weeks	-	10 (6–16)
Education		
<12 years	25 (3.1)	11 (6.3)
Completed high school	137 (17.0)	21 (12.0)
1–4 years higher education	334 (41.5)	53 (30.3)
>4 years of higher education	308 (38.3)	90 (51.4)
Tobacco use		
Used tobacco before pregnancy	90 (11.2)	-
Did not use tobacco before pregancy	714 (88.8)	
Use tobacco now	10 (1.2)	8 (4.6)
Does not use tobacco now	794 (98.8)	167 (95.4)
Country of birth		
Norway	620 (77.1)	113 (64.6)
Other	184 (22.9)	62 (35.4)
HDI birth country		
Very high HDI	710 (88.3)	134 (76.6)
High HDI	37 (4.6)	13 (7.4)
Medium HDI	23 (2.9)	11 (6.3)
Low HDI	30 (3.8)	17 (9.7)
Received information about iodine		
Yes	133 (16.6)	13 (7.4)
No	529 (65.9)	140 (80.0)
Do not remember	141 (17.6)	22 (12.6)

^a^ Values are presented as mean ± SD, median (p25–p75), and *n* (%). HDI: Human Development Index. ^b^ 20 missing from, height, weight, and BMI pregnant women. ^c^ 6 missing from pregnancy trimester.

**Table 2:** Personally perceived knowledge and confidence to achieve daily recommendations for iodine.

Iodine Knowledge	Pregnant Women (*n* = 804)	Lactating Women (*n* = 175)
	*n* (%)	*n* (%)
Do you know what iodine is		
No	202 (25.1)	12 (6.9)
Yes	414 (51.5)	103 (58.9)
Do not remember	188 (23.4)	60 (34.3)
I think I get enough iodine through the diet		
Agree	40 (5.0)	66 (37.7)
Disagree	274 (34.1)	7 (4.0)
Don’t know	490 (60.9)	102 (58.3)

**Table 3:** Knowledge regarding iodine’s dietary sources, functions, and national iodine status among pregnant and lactating women ^a^.

Iodine Knowledge	Pregnant Women (*n* = 804)	Lactating Women (*n* 175)
	*n* (%)	*n* (%)
Most important dietary iodine sources #		
Meat	126 (15.7)	24 (13.7)
Milk *	232 (28.9)	95 (54.3)
Fruit	85 (10.6)	28 (16.0)
Fish and seafood *	298 (37.1)	101 (57.7)
Bread	102 (12.7)	29 (16.6)
Vegetable oil	13 (1.6)	2 (1.1)
Iodized salt	331 (41.2)	81 (46.3)
Dietary supplements *	61 (7.6)	22 (12.6)
Don’t know	301 (37.4)	31 (17.7)
Iodine is important for #		
Normal child growth and development *	195 (24.3)	80 (45.7)
Prevent blindness	26 (3.2)	8 (4.6)
Normal fetal development *	131 (16.3)	30 (17.1)
Strength in teeth and skeleton	74 (9.2)	14 (8.0)
Maintain normal metabolism *	214 (26.6)	84 (48.0)
Prevent spina bifida	16 (2.0)	2 (1.1)
Don’t know	428 (53.2)	54 (30.9)
Iodine status in Norway #		
Too low intake is a current problem *	158 (19.7)	40 (22.9)
Too high intake is a current problem	26 (3.2)	0
Too low intake was a problem earlier, not now	63 (7.8)	15 (8.6)
Don’t know	569 (70.8)	119 (68.0)

* Correct answer, # Multiple answers possible.

**Table 4:** Iodine knowledge scores among pregnant and lactating women ^a^.

Iodine Knowledge Score	Pregnant Women (*n* = 804)	Lactating Women (*n* = 175)
Total score ^b^	7 (0–13)	12 (6–15)
None	208 (25.9)	18 (10.3)
Poor	177 (22.0)	28 (16.0)
Low	214 (26.6)	50 (28.6)
Medium	169 (21.0)	59 (33.7)
High	36 (4.5)	20 (11.4)

^a^ Values are presented as median (p25–p75) and *n* (%). Differences in score were tested with Mann–Whitney U test (*p* < 0.001). ^b^ Scores range from 0 to 24.

**Table 5:** Associations for being in the highest tertile of iodine knowledge score among pregnant and lactating women.

	Pregnant Women (*n* = 803)				Lactating Women (*n* = 175)			
	Unadjusted Coef.		Adjusted Coef. ^e^		Unadjusted Coef.		Adjusted Coef. ^e^	
	OR	95% CI	OR	95% CI	OR	95% CI	OR	95% CI
Education ^a^	1.63	1.20, 2.21 *	1.53	1.09, 2.14 *	1.49	0.78, 2.83	1.39	0.67, 2.91
Age	1.03	0.99, 1.06	1.02	0.98, 1.06	1.10	1.02, 1.20 *	1.14	1.04, 1.24 *
Number of children	1.01	0.82, 1.25	1.01	0.80, 1.27	0.80	0.54, 1.20	0.63	0.39, 1.01
Country of birth HDI ^b^	0.87	0.55, 1.39	0.82	0.51, 1.33	0.85	0.41, 1.79	0.66	0.26, 1.65
Information on iodine ^c^	2.85	1.95, 4.17 *	2.83	1.92. 4.17 *	2.77	0.89, 8.68	3.28	0.86, 12.55
Tobacco use ^d^	1.41	0.87, 2.30	1.13	0.68, 1.87	1.40	0.27, 7.14	1.15	0.20, 6.60

^a^ Categories for education: 0 = completed high school or lower education, 1 = higher education. ^b^ Categories for country of birth HDI: 0 = low/medium//high HDI, 1 = very high HDI. ^c^ Categories for information on iodine: 0 = none/don’t remember, 1 = yes. ^d^ Categories for tobacco use: 0 = use tobacco/did use before pregnancy tobacco, 1 = don’t use tobacco. ^e^ Adjusted for all variables in the model. Dependent variable: Knowledge score (0 < 66th percentile, 1 ≥ 66th percentile). * significant (*p* < 0.05).

**Table 6:** Differences in iodine status (UIC and BMIC) between knowledge scores above and below the 66th percentile among pregnant and lactating women ^a^.

	Knowledge Score < 66th Percentile	Knowledge Score > 66th Percentile	*p*
UIC pregnant women, µg/L (*n* = 777)	92 (59–140)	97 (62–150)	0.539
UIC lactating women, µg/L (*n* = 175)	65 (40–92)	64 (36–100)	0.791
BMIC lactating women, µg/L (*n* = 175)	66 (43–91)	76 (48–130)	0.097

^a^ Values are given in median (p25–p75).
